# Using science communication research to practice iterative engagement in collaborative nutrient management

**DOI:** 10.22323/2.23030801

**Published:** 2024-04-02

**Authors:** Katherine Canfield, Casey Chatelain

**Affiliations:** Environmental Protection Agency’s Office of Research and Development in the Atlantic Coastal Environmental Sciences Division in Narragansett, Rhode Island, USA. They are a qualitative social scientist by training, focused on applied research to improve stakeholder communication and engagement in water quality research. In the past her work has focused on inclusive science communication and justice in tourism development.; Environmental Scientist at Horsley Witten Group in Sandwich, Massachusetts. After graduating from College of the Holy Cross with a BA in Environmental Studies, she moved to Cotuit full time to work for Barnstable Land Trust. A lifelong sailor and a former commodore of the Cotuit Mosquito Yacht Club, Casey’s love for the water (especially Cotuit Bay) drove her to get a Masters of Oceanography from the University of Rhode Island.

**Keywords:** Environmental communication, Public engagement with science and technology, Public perception of science and technology

## Abstract

Thoughtful science communication is essential for the success of collaborative, transdisciplinary environmental research. We present an innovative evaluation of a four-year pilot project that took a highly engaged and collaborative approach to managing excess nutrients in the Cape Cod region of Massachusetts, USA. The evaluation approach included mid- and end-of-project interviews with researchers and project partners and a reflection from the lead science communication researcher. We found that an effective science communication evaluation needs to be (1) adaptive, (2) multistage, (3) holistic and objective-based, and (4) democratic and reflexive. Results demonstrate that formative and end-of-project science communication evaluation of research projects lead to improved engagement that better meets all collaborators’ needs.

## Introduction

Problem-solving is foundational to science, but often privileges the activities of researchers over consideration of the human impacts and communicating of solutions. These priorities are often overlooked in the research process. Collaborative projects that integrate science communication research, social and biophysical sciences, and practitioner knowledge provide opportunities to bridge research and practice approaches to problem-solving. Projects that seek to tackle multiple goals from multiple angles often use a transdisciplinary approach [Jantsch, 1972]. In addressing environmental challenges, transdisciplinary projects bring together professional scientific knowledge, practitioner knowledge, and others who bring different goals and have unique “stakes” to work towards societally beneficial environmental outcomes. All those with stakes in the project are considered “stakeholders,” regardless of being the team originating or funding the project, being an invited partner, or being someone otherwise impacted directly by the project’s outcome. In this practice insight, we use a holistic understanding of stakeholders, recognizing that collaborative projects appreciate diverse ways of knowing and various levels of stakeholder engagement.

The project evaluated here tackled the environmental challenge of nutrient management in a coastal watershed on Cape Cod, Massachusetts, USA. In this context, many diffuse “nonpoint” sources of pollution need to be managed rather than a single, concentrated source [Shortle, Mihelcic, Zhang & Arabi, 2020]. Because nutrient pollution does not originate from a single source, it requires researchers to collaborate with key stakeholders across several community and public sectors to address its spread from numerous sources [Patterson, Smith & Bellamy, 2013]. The project brought together stakeholders from federal, state, and local governments; universities; international and local nonprofits; and homeowners. Together, this team experimented with a suite of potential solutions to improve water quality with better nutrient management. This approach to stakeholder engagement and science communication research was novel for the U.S. Environmental Protection Agency’s (EPA) Office of Research and Development, the lead scientific organization on the project. To evaluate the effectiveness of this new approach to engagement, social scientists were engaged from the start of the project to its completion to capture the experience of the various stakeholders. These researchers used formative (mid-project) [Canfield, Mulvaney & Chatelain, 2022] and end-of-project data collection methods including autoethnography, content analysis, and semi-structured interviews with researchers and external stakeholders.

In this practice insight, we present the lessons learned about the strengths and weaknesses of using a two-stage, internal evaluation to analyze and inform a solutions-driven research process. The practice insight first discusses scientific literature on transdisciplinary collaborations and science communication evaluation. We then describe the methods used in our internal evaluation, including how the formative evaluation results were incorporated into the ongoing project. We next compare the results of the interviews held at the end of the project with those conducted mid-project [Canfield et al., 2022]. We finally discuss the value of internal two-stage evaluation, and lessons learned to improve future transdisciplinary projects using evaluation of science communication and public engagement with research.

### A brief overview of transdisciplinary collaboration

To achieve high-quality engagement in research, stakeholders must be accommodated to occupy various roles. These different roles allow for appropriate involvement across varied levels of effort, expertise, and priorities in a collaborative project. In our project, we prioritized high-quality participation, and, drawing on Shirk et al. [2012], we defined quality as how well a project’s goals, activities, and outputs align with those of the community and stakeholders. Prioritizing stakeholder engagement at various levels of intensity does not compromise scientific interests in transdisciplinary projects. Instead, it emphasizes the production of more equitable, usable results that better consider the needs of communities that have been marginalized in past scientific efforts [Cooper et al., 2021; Wilmsen et al., 2008]. A process and outcome that includes those at the margins will be more inclusive for all [Cooper et al., 2021; Ford & Airhihenbuwa, 2010].

Researchers have begun to consider how successful engagement in transdisciplinary projects can be measured. This expands the scope of what counts as a successful experiment, as it documents the various outcomes and outputs of collaborative projects that may not be captured through customary scientific measurements [Gross, 2006; Shirk et al., 2012]. The documentation of engagement and qualitative research complements quantitative research in transdisciplinary collaborations [Gross, 2006] and captures the specific place-based context of a project. In the context of managing excess nutrients in Cape Cod watersheds, this means appreciating that the region generally has low-density housing and large seasonal fluctuations in population. Technical solutions for the environmental challenge are important, but so too are supportive stakeholder relationships and ensuring that solutions align with the character and ways of life of the community [Bang, Marin & Medin, 2018; Mulvaney, Merrill & Atkinson, 2022; Stedman, 2003]. Another important marker of success that engagement research can capture is the relational and engagement experience of stakeholders throughout the project. While this may not be a direct measure of project success, poor relationships and communication will lead to less effective collaboration, and thus slow research progress. Engagement research can inform the process of building lasting collaborative relationships, which are essential to community-engaged research projects.

When integrating researcher and practitioner interests, expertise, and goals, science communicators must thoughtfully plan and execute activities. Early on, communicators need to identify communication and engagement goals for the project and map the different roles stakeholders will play based on their expertise, interest, and influence in the project [Rudman, Canfield, Mulvaney & Ridley, 2021; Weber & Backer, 2013]. Communicators need to tailor plans towards the specific actors and roles stakeholders will play in a project. This means considering the different relationships among collaborators and communities, and trust in science overall [Walls, Pidgeon, Weyman & Horlick-Jones, 2004]. For example, because a federal government agency was the lead organization on this nutrient solutions-driven research project, agency employees had to navigate the relationships the agency had with the community and project partners, and consider the perceptions these community members and partners had around trusting the government and science. To ensure successful and inclusive engagement when working with historically marginalized communities harmed by science or regulations, it is essential to actively consider actor perceptions and experiences [Callwood, Weiss, Hendricks & Taylor, 2022; Orthia, McKinnon, Viana & Walker, 2021]. Thoughtful communication planning requires a team member with skill and training in science communication. This person can prioritize activities that develop more trusting relationships throughout a collaborative project and ensure that engagement is not secondary to scientific experiments [Yuan, Besley & Dudo, 2019].

While science communication efforts for collaborative projects have increased, evaluation of the effectiveness of these efforts has not kept pace [Rowe & Frewer, 2004; Trench, 2014; Ziegler, Hedder & Fischer, 2021]. Like effective communication, those responsible for rigorous evaluation processes need to design and implement them from the start of a project [Jensen, 2014]. Quality (i.e., valid and complete) evaluations are challenging for evaluators to conduct in relatively objective ways [Rowe, Horlick-Jones, Walls, Poortinga & Pidgeon, 2008; Ziegler et al., 2021]. There is not an accepted framework used to evaluate science communication. The often context-specific nature of science communication makes developing a standalone framework challenging [Ziegler et al., 2021]. General frameworks for science communication evaluation [e.g. Garces et al., 2012; Rowe & Frewer, 2000; Revuelta, 2014] have not been extensively applied. Past studies have noted challenges in differentiating between goals and measurable objectives [Ziegler et al., 2021] and limited use of external (and assumed, less biased) evaluators [Esmail, Moore & Rein, 2015]. The extended timeline along which engagement efforts may occur also makes them challenging for evaluators [Esmail et al., 2015], especially if the research is bound tightly to the timelines of grant funding. Project members and evaluators may not find it straightforward to identify the end of a public engagement effort or relationship compared to completing a scientific experiment. Particularly lacking in science communication evaluation efforts to date have been formative (i.e., in-process or mid-project) evaluations [Esmail et al., 2015; Pellegrini, 2014]. This practice insight describes a two-stage science communication evaluation for a nutrients research effort on Cape Cod, Massachusetts, using both formative and reflexive data collection, providing a full-project view of communication quality.

### Case description

The Nutrients Solutions-Driven Research Pilot [EPA, 2023], a project based in the U.S. EPA’s Office of Research and Development, sought to experiment with both engagement and scientific methods. This collaborative project integrated researchers and practitioners across sectors and disciplines in New England with community-based stakeholders in leading problem identification and research prioritization. EPA researchers continuously engaged external stakeholders throughout the investigative process. We use the term solutions-driven research in this project to define the collaborative and transdisciplinary approach. This emphasizes partner-driven solutions in contrast to scientific curiosity-driven research.

This pilot focused on solutions to address nutrient pollution on Cape Cod. The main nutrient of concern in this case is reactive nitrogen, largely coming from minimally treated wastewater. Most wastewater is treated and disposed of onsite using conventional septic systems or cesspools, which are effective at mitigating bacteria but were not designed to remove nitrogen. This creates an oversupply of reactive nitrogen to nearby bodies of water, which can produce excessive amounts of algae and degrade habitat conditions for other organisms, including fish and shellfish. While not always a danger to humans, algae blooms are not aesthetically pleasing, and when harmful, lead to waterbody closures. On Cape Cod, a major destination for water-based recreation and tourism, such closures have large potential economic ramifications [Furey, Merrill, Sawyer, Mulvaney & Mazzotta, 2022; Merrill et al., 2018].

Some notable social and communication challenges complicate engagement with nutrient management on Cape Cod. One challenge is the “slow impact” of nutrient pollution [Canfield, Mulvaney & Merrill, 2021]. When excessive nutrients reach a surface waterbody through groundwater, they can cause observable impacts many years after addition of the pollutant has stopped [Carstensen, Sánchez-Camacho, Duarte, Krause-Jensen & Marbà, 2011; Van Meter, Basu, Veenstra & Burras, 2016; Merrill, Piscopo, Balogh, Furey & Mulvaney, 2021]. Along with the temporal disconnect between input and impact, the diffuse nature of nonpoint source nutrient pollution means there is also often a spatial disconnect between source and impact [Fowler et al., 2013]. This temporal and spatial distance makes it difficult for community members to see the roles they can play in helping to address this environmental challenge. Using local examples and presenting immediate actions that can be taken help address this mental distancing [Canfield et al., 2021]. Another challenge is communicating the importance of having enough nutrients, but not an excess, for healthy ecosystem functioning [Howarth & Marino, 2006; Nixon, 1993; Patterson et al., 2013]. Finally, navigating solutions and recommendations in the state of Massachusetts is challenging due to its governance as a commonwealth [Taylor, 2014]. In a commonwealth, regulations for wastewater management are decentralized such that they are largely determined at the municipal level rather than at the state level. Localized priorities in nutrient management become more important due to the increased influence of local government and the ability of town meeting attendees to vote on each decision [Perry, Smith & Mulvaney, 2020].

### Methods used

To evaluate stakeholder engagement experiences with this pilot solutions-driven research project, we used multiple qualitative methods both during and at the end of the project. We used formative [Canfield et al., 2022] and end-of-project data collection through autoethnography, content analysis, and semi-structured interviews with researchers and external stakeholders, focusing questions on evaluating engagement rather than the scientific aspects of the project (See questionnaire in supplementary materials). The first author, a social scientist focused on science communication research who was involved in the larger project on nutrient management, was the lead on data collection and analysis. She conducted all interviews over video calls and recorded and transcribed them to allow for accurate analysis. The social scientist was responsible for conducting all interviews to minimize the number of internal researchers included in the evaluation data collection.

**Figure 1.**
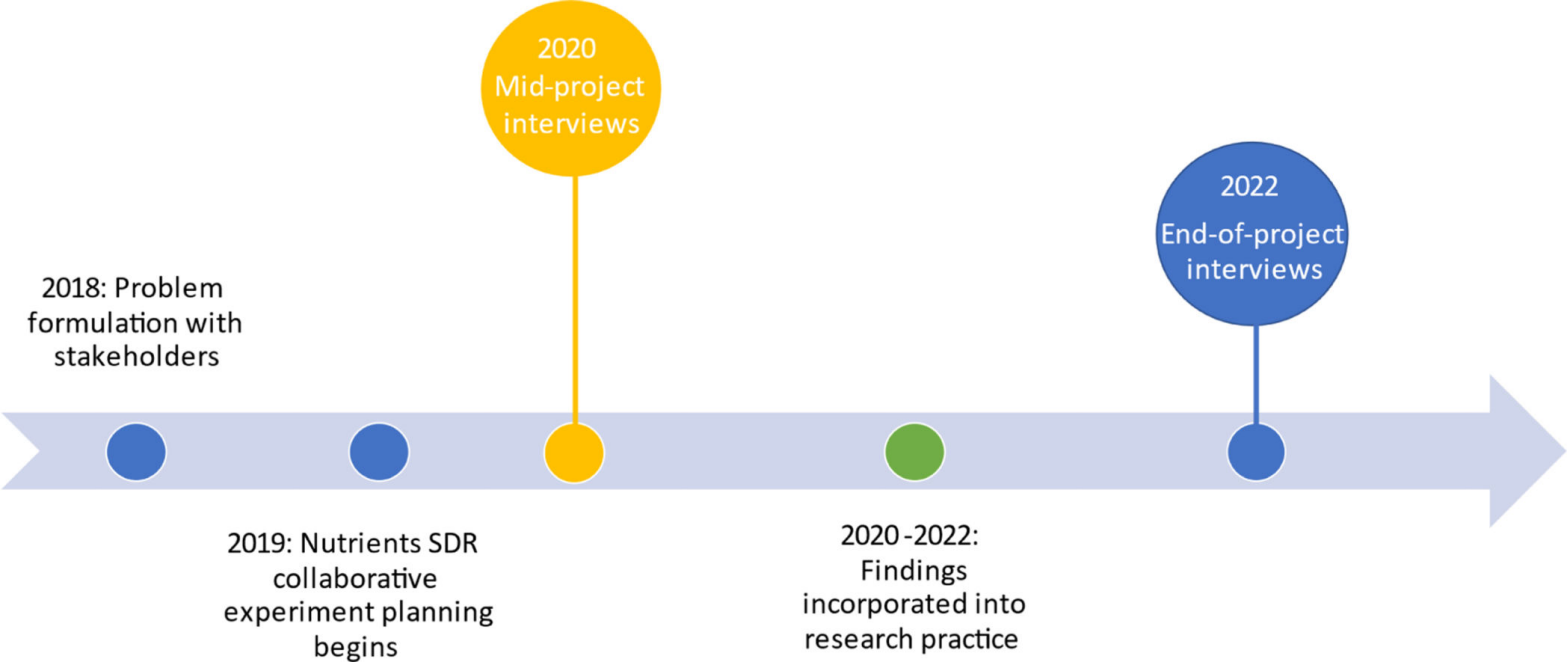
Timeline of Nutrient Solutions-Driven research mid-project and end-of-project evaluation.

We conducted interviews in the summer of 2020 and again in the summer of 2022 (Figure 1). In the first round of interviews, we interviewed all willing researchers on the EPA team (n=10), as well as external stakeholders coming from other federal government positions, nonprofits, universities, and local government (n=10). Due to the very specific population included in the evaluation, sociodemographic details about participants cannot be provided without compromising confidentiality. All participants or their replacements were included in the end-of-project evaluation through interviews in 2022. The only exception was one retiree without a replacement as of the time of the interviews (i.e., in 2022 there were nine EPA team interviews). Researchers designed questions that addressed communication and engagement goals that were set for the project at the end of 2019. Questions for researchers and external stakeholders varied slightly, mainly on questions that explored relational and inter-group dynamics. The initial interviews included some questions on past experiences and challenges with stakeholder engagement that were not included in the 2022 interviews, as they did not help to measure engagement efforts throughout the pilot. Researchers included all the other questions in the end-of-project interviews, with a few additional questions on how roles and engagement had shifted since talking last and whether identified markers of success had been achieved.

Using these interview transcripts, the first author analyzed how the innovative engagement and evaluation approaches were implemented in the research process. We analyzed all interviews using inductive qualitative coding in NVivo. This coding process involves looking for key themes within the data rather than working from a pre-identified set of categories in which to place responses. The initial codebook developed in analyzing the 2020 interviews for the formative evaluation [Canfield et al., 2022] was used as a starting point for the 2022 analysis. We added additional codes as new themes arose, and to categorize topics focused on experiences since 2020 (see supplementary materials for code list). Intercoder reliability checks involved another social scientist coding 20% of the interviews independently and then comparing results. We found at least 90% agreement between coders (similar to Floress, Kolozsvary and Mangun [2017]). Discussion of the discrepancies showed no meaningful difference in results. This check occurred for both rounds of coding to ensure reliable and consistent application of codes.

The two-stage evaluation also included data from an autoethnography [Holman Jones, 2007] and a review of engagement materials. As part of the formative evaluation, engagement materials produced throughout the pilot served as additional data to document varied efforts. Informal feedback from conversations and emails from both internal and external stakeholders on the project served to reinforce the use of effective engagement materials and shift efforts as appropriate. An autoethnography at the time of the second round of interviews served to collect the experiential perspectives of the lead author who led the interviews and the evaluation. The lead author conducted an autoethnography by documenting their responses to the questionnaire used with participants. The autoethnography provides both an additional perspective of researcher experience with the engagement process (as the lead author was also a part of the EPA pilot team) and a more holistic perspective on the engagement process due to expertise in science communication.

### Impacts of two-stage evaluation

Comparing perspectives collected partway through and at the end of the pilot project (in 2020 and 2022, respectively) revealed changes in roles and research priorities. In 2022, interviewees across stakeholder groups shared a perspective that the research focus had narrowed compared to the outset of the project. The main external partner’s interests received most of the funding and research focus. EPA researchers noted this as a novel experience of successfully aligning with collaborator priorities.

The shifting roles resulted in increased responsibilities for some and decreased responsibilities for others over time. Both researcher and practitioner collaborators had to adjust their involvement in the project based on how their research or expertise aligned with stakeholder participation and how project priorities changed from 2020 to 2022. Having two evaluation points revealed success in shifting priorities to align with collaborative goals, while also pointing out the need to plan for shifting responsibilities and stakeholder roles that result from such changes. Participants across sectors noted that clearly defined liaisons for each aspect of the project were missing. An external stakeholder shared, “I feel like we could maybe get a lot more done in terms of planning if we kind of know who all the different players are in this work off the bat.” Clarifying these liaisons could help to manage the increased responsibilities experienced among some participants. For participants with increased responsibilities, this also points to a need to ensure they are not overcommitted as the project evolves. Improving the experience of all collaborators in projects with evolving priorities will require setting clearer expectations and guidelines from the outset around adjusting time commitments and financial support.

In-process, or formative, evaluation is often overlooked in evaluating public engagement with science [Esmail et al., 2015; Ziegler et al., 2021]. By including in-process evaluation, the project team was able to employ adaptive engagement practices informed by the formative evaluation results. The evaluation results revealed that all collaborators valued stakeholder perspectives in research greatly [Canfield et al., 2022; Esmail et al., 2015]. Additionally, all collaborators perceived the diverse expertise of the large collaborative team as essential to the overall project’s success [Telford, Boote & Cooper, 2004; Vaughn & Jacquez, 2020]. As one participant noted, “I think working with every single partner has affected where this project is gone.” Mid-project interviews prompted shifts in communication approach that improved the experience for stakeholders. The addition of quarterly project updates with all interested community members was highly cited as effective at creating a more connected team.

Conducting evaluations at two distinct time points invited EPA researchers not used to consistent engagement in their research projects to reflect and participate in engagement continually and thoughtfully. Across researchers and external stakeholders, they noted higher rates of interaction with partners in the 2020 interviews compared to 2022. While some meetings at fixed intervals continued throughout the project, others observed a drop-off in communication between 2020 and 2022. EPA researchers noted, “it fell off towards the end,” and, “the last time I talked to you, I was probably more in the thick of engaging. . . and I didn’t do as much [engaging] lately.” This reduction was explained by the difference in communication needs between the early ideation, relationship building, and process planning stages and the later execution and analysis stages of the scientific experiments. The reduced communication across stakeholder groups as the experiments became more established emphasizes the specialized skills stakeholders brought to the collaboration. While check-ins still occurred, there was less need for clarifying roles once the experiments were underway.

Despite the perception among researchers of decreased communication, engagement materials and online meeting records demonstrate consistent engagement efforts throughout the project. The autoethnography results show that much of the written and printed communication products were the effort of a small number of SDR project participants, often in support of larger teams’ scientific work. Likely, researchers did not consider the communication products being shared with external stakeholders that were about projects and communication efforts beyond their own in estimating the amount of communication and engagement materials being shared. Based on the review of engagement materials, communication with external stakeholders and community members continued at a consistent rate throughout the project. An additional likely explanation for the change in perceived meeting frequency was the ongoing COVID-19 pandemic. Mid-project interviews occurred early in the pandemic, not long after extensive in-person meetings occurred. In-person meetings were significantly less frequent after March 2020, although virtual meetings continued throughout the project. While the second evaluation occurred at the formal end of the pilot project, communication efforts will likely continue as scientists publish results and external stakeholders continue nutrient management work in their communities.

### Lessons from evaluation

**Figure 2.**
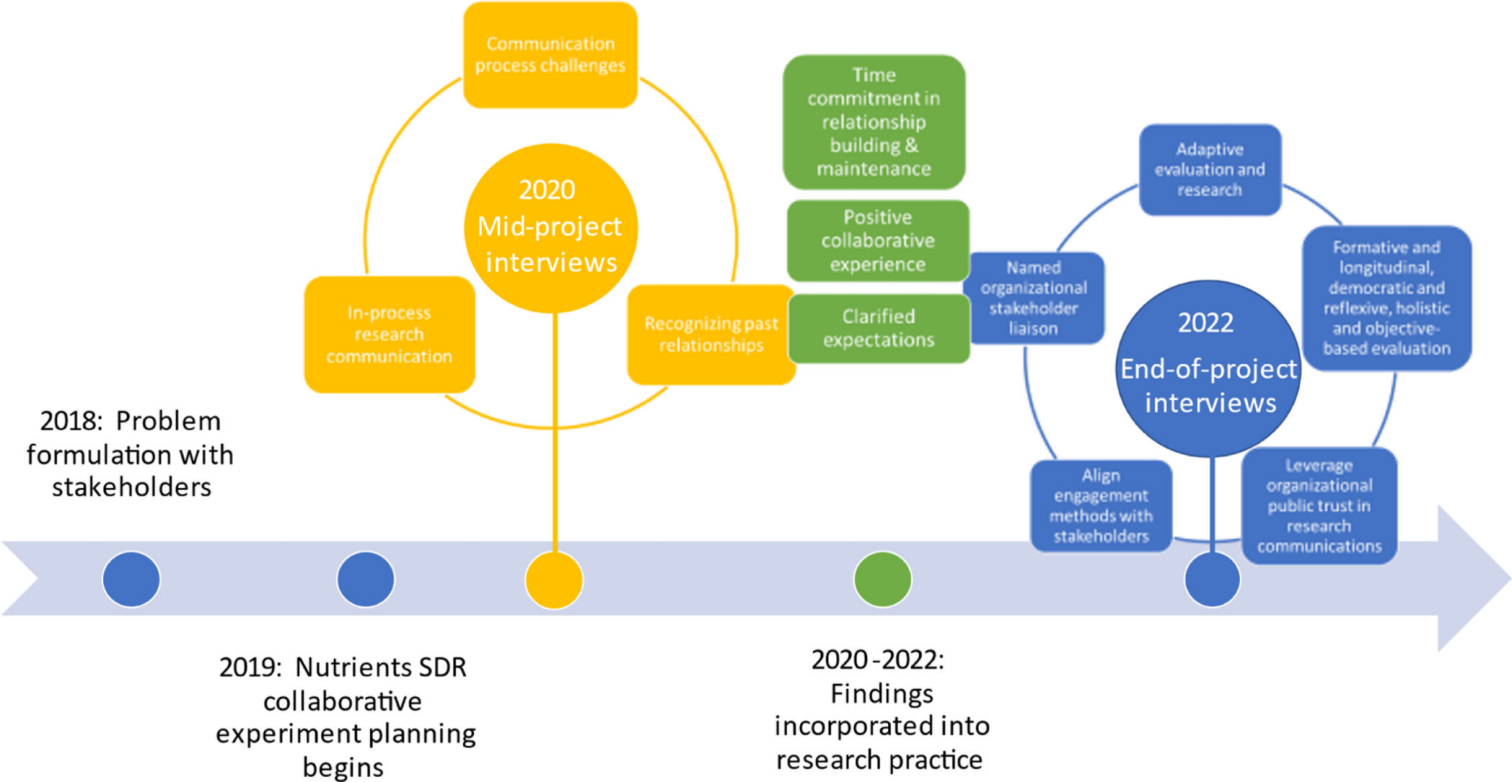
Lessons learned from the mid-project and end-of-project evaluation of the nutrients solutions-driven research pilot. Lessons in yellow were exclusive to mid-project evaluation, and blue were exclusive to the end-of-project evaluation. Listed lessons in green were observed in both rounds of interviews.

The results of this evaluation identified a few key lessons in a two-stage evaluation approach for community-engaged research projects (see Figure 2). The formative evaluation identified the novelty for scientific researchers of engaging in science communication at the EPA during the research process and identified several ways to improve the ongoing project. EPA researchers repeatedly mentioned, “We did more engaging with our stakeholders from the get-go and more conversations about what their needs were and how to make this a better project for them.” Including the end-of-project evaluation allowed us to document how the project team incorporated early recommendations and how relationships and research evolved. Importantly, having data from these two time points with largely the same participants allowed for documenting how interviewees’ perspectives on and recommendations for solutions-driven research changed from before to after implementing experiments. Both formative and end-of-project interviews were essential for us to evaluate how continued engagement in a transdisciplinary project confirmed early perspectives on the significant amount of time commitment required to build and maintain relationships, the positive experience of collaboration, and the need to clarify expectations. In the end-of-project interviews, interviewees felt less strongly about the challenges associated with sharing results. This change points to both the progress in navigating existing communication processes to share results and how the conditions at the time of an interview influence interviewees’ primary concerns. Additional lessons that resulted from having participants reflect on their experience at the end of the project include the need to (1) leverage community-based organizations for public trust in research communications, (2) vary engagement methods and venues to align with varied stakeholder perspectives, (3) adapt to evolving stakeholder roles and priorities, and (4) have a named liaison for stakeholder engagement within each organization to reduce the effort required to build connections.

An early success of this evaluation was the integration of recommendations from the formative evaluation (2020) into the project. Through leaders’ adaptive management as the project progressed, shifting engagement approaches allowed research to be shared more effectively with collaborators and interested community members. Research was shared effectively through continued biannual bulletins on all aspects of the project and bimonthly to quarterly meetings featuring updates from both EPA researchers and external partners. Each meeting gave updates on a distinct aspect of the project (e.g., septic tank experiments, cranberry bog restoration, or harmful algal blooms) and left ample time for questions from interested stakeholders and community members. One EPA interviewee noted, “We’ve had a lot of engagement with the state through that vehicle. I think that’s another important mechanism and that helps with the engagement, but it also helps with conveying the science.” We recommend conducting formative evaluations, as they encourage continued consideration of how collaborators engage with the project and allow for data-backed improvements to the process.

Another lesson for future evaluations is to create a mechanism to evaluate the overall project rather than just the communication and engagement efforts. While there were communication and engagement goals identified early on, the overarching end goals for the project remained unclear, which led to limiting the evaluation to focus on communication and engagement [Canfield et al., 2022]. Best practices in science communication encourage the identification of quantifiable objectives that are distinct from the engagement goals. The team did identify metrics to measure progress towards engagement goals and instituted tools for tracking, but inconsistent participation in collecting this data made it of limited use for evaluating progress. Getting buy-in from those responsible for contributing data to tracking mechanisms is essential to create a more rigorous evaluation process.

The value of democratic evaluation is a final lesson for effective evaluation. Including stakeholders across various sectors in the evaluation process allowed for multidirectional feedback, and stakeholders perceived more shared responsibility and roles than they expected in a government agency collaboration. Interviewees consistently mentioned trust across organizations, and EPA researchers spoke of the value of working closely with a trusted local organization to speak with members of the community in which the experiments were occurring. Having an early-career researcher conduct the evaluation interviews also proved to encourage honest participation and reduce power dynamic concerns [Anyan, 2013] about sharing perspectives that could be perceived as critical of the agency.

**Figure 3.**
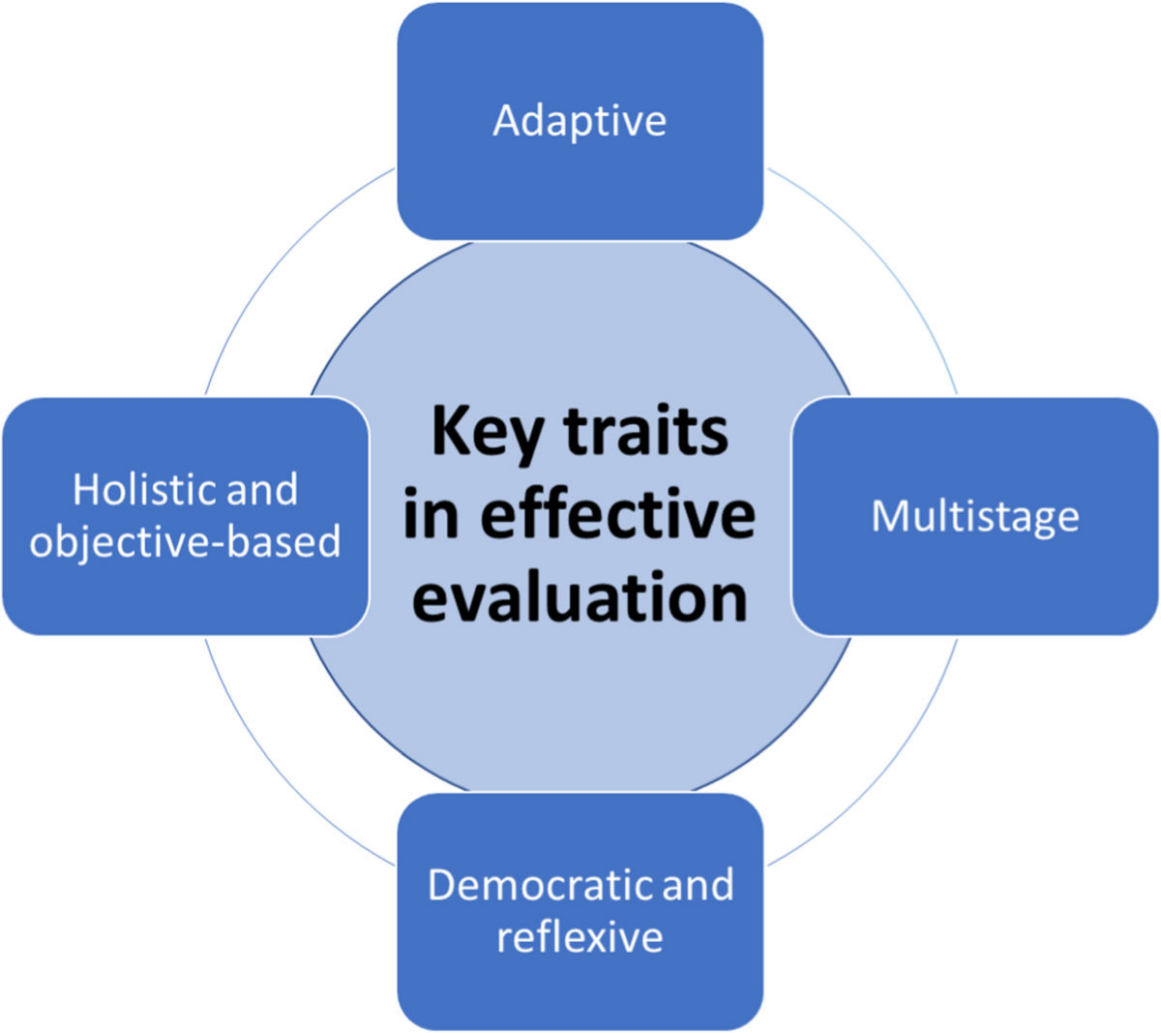
Key traits in effective evaluation based on a two-stage evaluation of a transdisciplinary project.

Together, these lessons demonstrate that evaluating an evaluation is another important step in effective collaborative processes and can reveal areas for improvement in future transdisciplinary research efforts. We identify key traits of an effective science communication evaluation as being adaptive, occurring at multiple time points in the project (i.e., multistage), being democratic and reflexive, and being holistic and objective-based (Figure 3). In evaluating this evaluation on these four key traits, we identified numerous lessons that can be carried forward to improve future evaluations of transdisciplinary projects. Broadening the evaluation to evaluate the project overall (beyond just communication and engagement) will improve holistic understanding of project strengths, changing priorities, and challenges, and will improve adaptability. To make the evaluation more objective-based and improve the quality of the two-stage evaluation points, increased effort should be invested in communicating the importance of, and ensuring consistent participation in, collecting data on communication and engagement efforts. Relatedly, future evaluation efforts should also focus on getting buy-in from those responsible for contributing said data and simplifying data submission to encourage participation. For a project lasting longer than the one studied here (i.e., more than four years), it would be valuable to add additional stages of evaluation to have data and iteratively improve engagement throughout the project. Based on the experiences of this project, we would also recommend extending our collection of communication and engagement data after the end of the project, changing from a two-stage to a multistage evaluation. Along with providing an additional stage of evaluation, this would improve understanding of the evolution of collaborative relationships, community capacity to maintain projects, and longevity of research outcomes. Learning from the successes of this approach, future evaluations should continue integrating early evaluation results into the ongoing project, and mid-project evaluation results should influence the structure of the end-of-project evaluation. Finally, to ensure a more democratic and reflexive evaluation, future projects will also need to continue to consider the power dynamics between the evaluator and participants to ensure trust and honesty.

There are notable limitations to conducting an internal evaluation of a research project [Rowe et al., 2008; Ziegler et al., 2021]. The lead researcher’s role as a member of the EPA pilot team, though new to the organization, reduces objectivity in analyzing the effectiveness of engagement efforts. Despite these shortcomings, the use of a consistent evaluation tool to measure distinct stakeholder groups’ experiences with engagement during and at the end of the project aligns with recommended practices for science communication evaluation. Further, these evaluation methods allowed for noting both the successes of innovative engagement in research and areas for improvement in future efforts [Ziegler et al., 2021]. As a social scientist designed and executed the evaluation at the outset of their involvement with the project, the evaluation was a rigorous scientific investigation rather than an afterthought.

## Conclusion

This practice insight presents how a two-stage evaluation effort can help bridge the researcher-stakeholder divide in transdisciplinary research by encouraging adaptive engagement. Key engagement lessons learned from the multimethod approach included the importance of clearly defining expectations, continuous targeted engagement, investment in relationship building and maintenance, and specifying liaisons between stakeholder groups. We also found that timing of an evaluation will impact how participants perceive the current successes and challenges in a project. This further points to the value of multistage evaluations in identifying the themes that carry across evaluation stages, including beyond conclusion of a project, to better characterize overall experiences with a project. As such, we identify the key traits of effective science communication evaluation as being (1) adaptive, (2) multistage, (3) holistic and objective-based, and (4) democratic and reflexive. Together, these findings can improve our evaluation of transdisciplinary projects as well as the experiences of all collaborators involved.

## Supplementary Material

SI
