# Supplementary material for: Using science communication research to practice iterative engagement in collaborative nutrient management
Source: JCOM J Sci Commun. Author manuscript; Available in PMC 2025 Apr 2. (PMC11235194; doi:10.22323/2.23030801)
Supplement: SI [file NIHMS1991671-supplement-SI.pdf]

Canfield, K. and Chatelain, C. (2024). ‘Using science communication research to practice iterative engagement in collaborative nutrient management’.
JCOM Supplementaty Material

Questionnaire used for interviews with researchers and stakeholders

Key: *Italics*: asked only in 2020; 1 asterisk (*): asked in 2020 and 2022; 2 asterisks (**): asked only in 2022

Questions for SDR Researchers

- How do you think your research is beneficial to the communities on Cape Cod?*
- *What do you see as your role in the nutrients SDR pilot?*
- Do you think your role in the nutrients SDR pilot has shifted since 2020?**
- What do you see as markers of success for your research in this project?*
  - Do you think you’ve achieved them?**

General EPA Communications

- *Have you interacted with the EPA communications people (define who they are)? How frequently?*
- *What experience do you have with EPA communications people?*
  - What have been the barriers to publishing communication materials?*
  - *Have you found any of the processes particularly straightforward (or notably convoluted)?*
- *What experience do you have with public-facing communication products beyond peer-reviewed articles?*
  - *Have you made a palm card, fact sheet, or written a science matters article?*
- Over the past two years, have you had any new experiences with EPA communications people?**
- Over the past two years, have you had any new experiences with making public-facing communication products beyond peer-reviewed articles?**

Stakeholder Engagement in Research

- What type of products/formats/activities do you think of as stakeholder engagement?*
- *What kind of stakeholder engagement has been a part of your past work prior to this pilot?*
  - *What motivated you to engage with stakeholders in past projects?*
- *What kind of impediments have you experienced in trying to engage with stakeholders in your past research?*
- *How have you previously identified relevant people to engage?*

Nutrient SDR engagement

- How does this project’s approach to engagement differ from past projects?*
- Which stakeholders have you personally interacted with the most for your research on this project?*
  - How frequently do you interact with these stakeholders?*
  - Are you satisfied with the contributions from your stakeholders?*
  - Are there areas where there could be better quality/frequency of contributions?*

- How has your team/aspects of the project benefitted from working closely with stakeholders (overall/for your team specifically)?*
    - Any specific examples?*
    - What are the drawbacks?*
- What kind of impediments have you experienced in trying to engage with stakeholders in your research for this pilot?*
- *How do you think stakeholders should be involved in the Cape Cod project overall?*
    - *How about for your specific project (should they be more or less involved)?*
    - *A variety of stakeholders are currently a part of the project in many different ways – do you think this is adequate (to reach project/your research goals)? (Is it excessive?) If not, what might need to change for adequate stakeholder involvement across various relevant groups?*
- Why do you think stakeholder engagement has been a part of this project (overall/for your team specifically)?*
    - Whose job is it to engage with stakeholders for this project? Why?*
    - What forms of engagement do you think have been most effective? Why?**
- Has engagement with stakeholders changed how you did your research? How?*
- Has stakeholder engagement affected your anticipated outputs/the products you're making? How?*
- As a percent of your overall research time, how much of that time has been focused on this nutrients pilot? Of that time, what percent of your time do you dedicate to stakeholder engagement?*

Solutions-Driven Research at EPA and Moving Forward

- How would you define (describe) solutions-driven research?*
- Based on your experience with this pilot, what do you think about expanding EPA's application of a solutions-driven approach to research?*
    - Does it make sense to expand this approach to apply it to the majority of ORD's projects?*

Lessons learned

- What would you differently next time in engaging with stakeholders?*
    - Are there ways you could improve how you engage with stakeholders? *
- What have you learned from the SDR pilot that will shape your efforts to engage with stakeholders moving forward?*
    - Are there any tools/trainings that would have made you feel more comfortable with stakeholder engagement?*
- Do you have any advice on how to improve ACESD's/ORD's approach to solutions-driven research based on your experience with this project?*
- Anything else you'd like to add about your experience with stakeholder engagement in the solutions-driven research pilot?*

Questions for stakeholders

- What do you see as your role in the nutrients SDR pilot?*
    - Has that evolved in the past couple of years**
- How do you think EPA's research is beneficial to the communities on Cape Cod?*
- What do you see as EPA's role in the nutrients SDR pilot?*
- What do you see as markers of success for your work on this project?*
    - Do you think we've achieved success in this way?**

Stakeholder Engagement in Research

- What type of things do you think of as stakeholder engagement?*
- *What kind of stakeholder engagement/inter-organization collaboration has been a part of your past work prior to this pilot?*
- *What kind of impediments have you experienced in trying to engage with stakeholders (researchers) in your past work?*

Nutrient SDR engagement

- Why do you think stakeholder engagement is a part of this project (overall/for your team specifically)?*
- How does this project's approach to engagement differ from past projects?*
- Which stakeholders (researchers) have you personally interacted with for this project?*
    - How frequently do you interact with these stakeholders?*
    - Have you worked with these stakeholders before?*
    - Are you satisfied with the contributions from these stakeholders?*
    - Are there areas where there could be better quality/frequency of contributions?*
- How has your organization benefitted from working closely with EPA on your work in this project (overall/for your team specifically)?*
    - Any specific examples?*
    - What are the drawbacks?*
- What has gone particularly smoothly in working with EPA on this project?*
- What kind of impediments have you experienced in trying to engage with EPA and other stakeholders for this pilot?*
    - What has been difficult about working with EPA on this project?*
- Are there any notable challenges to working with EPA that go beyond just completion of the research?*
- What do you think of the balance of roles played by EPA and other stakeholders in the project overall?*
    - *How about for your piece of the project (should they be more or less involved)?*
    - *A variety of stakeholders (including researchers) are currently a part of the project in many different ways – do you think this is adequate (to reach project/your research goals)? (Is it excessive?) If not, what might need to change for adequate stakeholder involvement across various relevant groups?*
    - Whose job is it to engage with stakeholders for this project? Why?*
- What forms of engagement do you think have been most effective? Why?**
- Has engagement with EPA changed how you proceeded with this project? How?*
    - Has stakeholder engagement affected the anticipated research products you're making? How?*

- As a percent of your overall work time, how much of that time has been focused on this project? Of that time, what percent of your time do you dedicate to stakeholder communications?*

Lessons learned

- What would you do differently next time in working with EPA?*
  - How might that have improved the project?**
- What do you think EPA should do differently next time in building a research partnership? In engaging with researchers/stakeholders?*
- What have you learned from this project that might shape your efforts to engage with research institutes or EPA moving forward?*
- Do you have any advice on how to improve ACESD's/ORD's approach to solutions-driven research based on your experience with this project?*
- Anything else you'd like to add about stakeholder engagement in solutions-driven research?*

Nutrient SDR engagement codebook

Name	Description
1. EPA research beneficial	General node in which to house various ways EPA research is described as beneficial to Cape Cod communities
communication tools	
funding - stakeholders view	
identify effective nutrient solutions	
stakeholders agree	
policy process info	
restoration support	EPA research is beneficial to cape communities in supporting restoration projects in the state
scientific expertise	EPA research beneficial to Cape Cod communities for providing specialized experience in scientific research
stakeholders agree	
stakeholder connections	EPA partnership beneficial to Cape Cod communities by providing connections to various potential partners in the community
1. Markers of success	what counts as success in the SDR pilot? This is a parent code for the various responses
alt. comm products	Researchers' willingness to communicate their work in ways beyond peer-reviewed papers as a marker of success of SDR
experiments happening	
stakeholders agree	
success achieved	

Name	Description
success achieved OR in-progress	considered their work a success as of interview
in-process comm. +eval.	Success as keeping track of engagement efforts/communicating research while in process
stakeholders agree	
success achieved or in progress	
internally beneficial	
success achieved or in progress	
long-term + transferrable results	experiments providing results that can be transferred to other cases
stakeholders agree 22	
success achieved	
N reduction	
stakeholders agree	
success achieved	
peer-reviewed research	
success achieved OR in-progress	
SDR seen as beneficial	A marker of success for the project being SDR approach being seen as beneficial across ORD
stakeholder use	

Name	Description
the science	
stakeholders agree	
success achieved or in progress	
trust and relationship building	
success achieved or in-progress	
2.0 EPA comm office	Parent node for comments about EPA communication staff interactions
2022 new experiences	
2022 no new experiences	
clearance challenges	difficulty with the timing and effort related to getting products cleared through EPA comm processes
lack of process clarity	
timing	
design	
failure to communicate w researchers	challenge of there being little understanding of EPA research among comm staff
limited ability to produce targeted comms	
motive for interactions	mentions of why they were working with EPA comm/public affairs staff
positive processes-experiences	

Name	Description
2.1 Comm products - not papers	
2.3 Kinds of engagement	Parent node for descriptions of different kinds of stakeholder engagement, and how they have been involved for the SDR pilot
bimonthly meetings - not inclusive	
chats in the field	
defining stakeholder engagement	interviewee definitions of what counts as stakeholder engagement
stakeholder defn	
emails and phone calls	
field example pres.	
presentations	
research logistics support	
sci support	
small group meetings	
town or community meetings	
workshops	
written - bulletin etc	
2022 - change over time	
personnel changes	
reduced	

Name	Description
engagement over time	
Role change by 2022	Parent node for comments on how roles changed in the project over time
bigger role 22	
focus shift	
Same role 2022	
Smaller role as of 2022	
Expertise not needed	
3.0 Difference of SDR pilot	parent node for descriptions of how this pilot research has been different than past work
early engagement w stakeholders	
forced engagement	
hierarchical influence of partners	some stakeholders have more power than others in this pilot
more internal comm	
social science integration	
stakeholder perspectives	
consistent comm w stakeholders	
stakeholder perspectives- not diff	

Name	Description
stakeholder perspectives- yes different	
3.1 Key stakeholders	
frequency of engagement	
internal stakeholder challenges	Challenges with engagement due to EPA stakeholder relationship/timing of response
more engagement desired	
satisfied with stakeholder contributions	
funding	
stakeholders' connections 22	
stakeholders EPA connections	Who at the EPA are external stakeholders are seeing as close connection
3.2 Stakeholder impact on research	How have stakeholders and engagement impacted the outputs of the project?
constraining topics to be pursued	
experiential knowledge	
experiment design	
interpretation of results and revised products	working closely with stakeholders adjusting the application of research - influencing the scale and perspective for which results are interpreted
no impact	
positive collaboration	
scale of research	

Name	Description
3.3 Drawbacks	parent node for descriptions of drawbacks of the pilot approach
don't understand social science	
EPA stigma	stigma about working with the EPA
keeping everyone on same page - communication	
location	
meeting partner needs	lack of objectivity, meeting diverse stakeholder needs
none	
research focus doesn’t match scientists’ goals	
time commitment	
3.4 Why stakeholder engagement	
How to engage for nutrient SDR	
stakeholder view	
3.5 Whose job is engagement	
stakeholders' perspective	stakeholders' view of whose job engagement is
3.6 Effective engagement approaches	Parent node for most effective engagement in project as of 2022
Bulletins	
diversity of approaches	
high frequency	

Name	Description
presence at field sites	
quarterly meetings	
research design-site selection	
small group exchanges	
in-person	
workshops	
3.7 percent time committed to project	
4.0 Defining solutions-driven research	comments that either directly elicit or indirectly result in describing the pilot approach as driven by community needs
SDR approach is not new	The stakeholder-driven, problem-solving focus of SDR research being described as something familiar or "not novel"
4.1 Expanding use of SDR approach	
do not expand to be majority of EPA research	
need folx w skills in translation	
4.2 Areas for improvement in future sdr	recommendations for how to improve EPA efforts at SDR
choose right partner	
choose EJ community partner or locale	
collab-comm w other SDR projects	

Name	Description
comm approach	
comm planning earlier	Develop communication plans earlier in future SDR projects
comm templates	
follow-up workshops on project status	further workshops that let all stakeholders know what's going on
improved in-process communicatio n	
follow thru to END of project	
iterative problem formulation	
less strict approaches to engagement	
more inclusive project formulation	
more researcher interaction w stakeholders	
finances	
budget transparency	
compensating partners	
haphazard management	

Name	Description
more trainings - pro and con	comments both supporting and opposing further training to improve SDR efforts
relevant policy for project	
social sci background training	a training on the methods and approaches of social scientist
stakeholder engagement and translation	
workshops	
prof credit for engagement	
roles and responsibilities	discussion of problems with the management of roles and responsibilities
clarify comm responsibilities	
clearer management structure	
keep more roles in EPA ORD	
navigating decreased need for expertise	
standardizing SDR	EPA-scale process improvements related to how SDR should be done
clearer bounds of EPA ORD roles	
relationship maintenance	
4.3 Learned from SDR Pilot	

Name	Description
clarified ORD role in SDR problems	
engage early and often	
face to face importance	
gov. partner preferred	
managing expectations	
more comm products for stakeholders	
other	
social sci as community engagement	
time commitment for engagement	
5.1 Stakeholder role in pilot	
5.2 Stakeholder view of EPA role	
comm and sharing results	
legitimacy	
project management	
scientific expertise	
5.3 Stakeholder view satisfaction w contributions	
need more stakeholder	

Name	Description
involvement	
5.4 Stakeholder view - benefit of EPA AND impact on research	
broadening topics pursued	
community-focused research	
epa gives credibility	stakeholder perspective that working with EPA give credibility to a project
funding	EPA makes it so that partners don't have to worry about funds
momentum+ pace of progress	positive experience with the pace of research/installing experiments
positive collaboration and networking	
positives of sdr - letting stakeholders lead research topics	
provides transferrable results	
research+scientific expertise	
9. Stakeholder critiques+ recs 4 improvement	stakeholders recommendations of how to improve engagement on the project and challenges in working with EPA
EPA bureaucracy	comments about EPA bureaucracy and rules
comm bureaucratic challenges	
EPA's role - clarify	
increase EPA ctrl,	

Name	Description
reduce stkholder ctrl	
keeping big pic clear	Losing track of what the big picture goal is has been a challenge
more full stakeholder comm meetings	
more translated science	science says very technical in comm products - need to make it more accessible
not enough labor focused on program management	
Stakeholder roles clearer	
timing	comments about how the project is going slower than they'd like
work through existing relationships	
9. Stakeholder lessons learned	
collaborative working w partners	
less fear talking to experts	
make working groups sooner	stakeholder learned the value of making project-specific working groups earlier in the research process
need for baseline data	
strategic engagement	learning a more involved, planned approach to engagement
Critiques of engagement	Commentary on what methods of engagement are more or less effective, and why
missing	

Name	Description
stakeholders	
no follow-up	
pandemic challenges	
Past stakeholder engagement experience	Interviewee descriptions of past stakeholder engagement efforts
obstacles to engagement	
diff research and decision-making priorities	
fear of talking to regulatory agency	Stakeholders' fear of engaging w EPA because of potential regulatory response
funding and proposal rules as EPA researchers	
time commitment of researchers	
understanding community needs	struggle to ID community needs
Pilot building on past research	Current SDR pilot is building on past research on Cape Cod that built relationships and potential research topics
Pilot project overall positives	
Positive engagement feedback	
responsive to stakeholder needs	
trust and relationship building	examples of when stakeholder relationships have become more trusting, or there is collaborative give and take to the relationship
